# Prenatal COVID-19 Exposure and Neurodevelopmental and Neurological Disorders in Children

**DOI:** 10.1001/jamanetworkopen.2026.31847

**Published:** 2026-09-03

**Authors:** Maria C. Magnus, Helena N. Eide, David P. Burgner, Olof Stephansson, Siri Eldevik Håberg

**Affiliations:** 1 Centre for Fertility and Health, Norwegian Institute of Public Health, Oslo, Norway; 2 Department of Infection Control and Preparedness, Norwegian Institute of Public Health, Oslo, Norway; 3 Department of Paediatrics, The University of Melbourne, Parkville, Victoria, Australia; 4 Murdoch Children’s Research Institute, Royal Children’s Hospital, Parkville, Victoria, Australia; 5 Faculty of Health, Deakin University, Burwood, Victoria, Australia.; 6 Clinical Epidemiology Division, Department of Medicine, Solna, Karolinska Institutet, Stockholm, Sweden; 7 Department of Women’s Health, Karolinska University Hospital, Stockholm, Sweden; 8 Department of Global Public Health and Primary Care, University of Bergen, Bergen, Norway

## Abstract

**Importance:**

Previous studies suggest that children exposed to COVID-19 during pregnancy may have an increased risk of neurodevelopmental disorders. However, these studies had limited follow-up periods and primarily evaluated broad neurodevelopmental concerns.

**Objective:**

To examine whether exposure to maternal COVID-19 during pregnancy is associated with an increased risk of neurodevelopmental or neurological disorders among offspring.

**Design, Setting, and Participants:**

This cohort study was conducted between November 2025 and February 2026. The sample included all children born in Norway between March 1, 2020, and December 31, 2023, with 22 completed gestational weeks and a birth weight greater than 500 g. Data were obtained from national registries, including maternal background characteristics from Statistics Norway, birth characteristics from the Medical Birth Registry of Norway, prenatal exposure to COVID-19 from the Norwegian Surveillance System for Communicable Diseases, and neurodevelopmental and neurological disorders from the Norwegian Patient Registry.

**Exposure:**

Maternal COVID-19 during pregnancy, which was defined as a positive PCR result for SARS-CoV-2 infection between conception (date of birth minus the gestational duration in days) and the date of delivery.

**Main Outcomes and Measures:**

The 8 outcomes of interest were any neurodevelopmental disorders, pervasive neurodevelopmental disorders (autism), developmental speech or language disorders, developmental motor disorders, intellectual disorders, any neurological disorders, epilepsy, and sensory impairments. Diagnoses of neurodevelopmental disorders, neurological disorders, and sensory impairments were registered in the national patient registries. Risks were evaluated using Cox proportional hazards regression.

**Results:**

Among the 204 663 children (104 728 males [51.2%]) included in this study, 16 325 (8.0%) were exposed to maternal COVID-19 during pregnancy. Exposed children did not have increased risk of any neurodevelopmental disorders (adjusted hazard ratio [AHR], 0.97; 95% CI, 0.88-1.08), pervasive neurodevelopment disorders (AHR, 0.88; 95% CI, 0.67-1.15), developmental speech or language disorders (AHR 1.13; 95% CI, 0.80-1.59), developmental motor disorders (AHR, 0.83; 95% CI, 0.44-1.56), intellectual disorders (AHR, 1.19; 95% CI, 0.65-2.19), any neurological disorders (AHR, 0.97; 95% CI, 0.88-1.07), epilepsy (AHR, 1.14; 95% CI, 0.87-1.48), or sensory impairments (AHR, 1.09; 95% CI, 0.92-1.30). These findings were mostly robust across sensitivity analyses and did not vary according to trimester of exposure or the dominant circulating SARS-CoV-2 variant. The exception was an increased risk of speech or language disorders among exposed children, excluding children born after March 1, 2022 (AHR, 2.22; 95% CI, 1.44-3.41); this sensitivity analysis was performed to limit misclassification due to reduced availability of SARS-CoV-2 testing.

**Conclusions and Relevance:**

In this population-based cohort study, children exposed to maternal COVID-19 prenatally did not show an increased risk of neurodevelopmental disorders, aside from an increased risk of speech or language disorders among those exposed in the early phase of the pandemic. These findings should be replicated in other populations and settings.

## Key Points

**Question:**

Is exposure to maternal COVID-19 during pregnancy associated with an increased risk of neurodevelopmental or neurological disorders among offspring?

**Findings:**

In this cohort study of 204 663 children, no associations were found between maternal COVID-19 in pregnancy and neurodevelopmental outcomes in offspring. However, the analyses restricted to the period with the lowest risk of misclassification of infection indicated that children exposed to maternal COVID-19 had an increased risk of developmental speech or language disorders.

**Meaning:**

The findings warrant additional examination into the risk of speech or language disorders among children exposed to prenatal COVID-19.

## Teaser

This cohort study explores the risk of neurodevelopmental or neurological delays among offspring who were exposed to maternal COVID-19 in utero.

## Introduction

Both human data and animal models indicate that immune responses during pregnancy impact fetal brain development.^1,2,3,4^ SARS-CoV-2 infection in pregnancy results in maternal and placental immune responses,^5,6,7,8,9^ and such immune responses may affect neuronal development and function, synaptogenesis, and axonal targeting.^10^ Offspring of mothers with SARS-CoV-2 infection during pregnancy have an increased risk of fetal death (stillbirth and miscarriage), preterm birth, and intensive care in the first weeks of life.^11,12,13,14^

Studies investigating neurodevelopmental outcomes among children exposed to COVID-19 in pregnancy have mostly examined neurodevelopmental scores^15,16,17,18,19,20,21^ or clinical diagnoses,^22,23,24^ with most restricted to the first 2 years of life. The findings have been inconclusive, with some studies reporting worse neurodevelopment,^18,19,22^ while others have reported no robust evidence of an increased risk of these outcomes.^15,16,17,20,21^ A more recent study with follow-up to 3 years of age reported increased scores on specific domains of neurodevelopmental diagnoses or autistic disorder among offspring exposed to maternal COVID-19 during pregnancy, with an adjusted odds ratio (AOR) of 1.29 (95% CI, 1.05-1.57).^22^ The largest study to date on diagnosis of autism among offspring exposed to maternal COVID-19 during pregnancy (n = 69 987) suggested that an increased risk might only be observed among female offspring.^25^ There is a lack of studies on neurological disorders, including epilepsy, among children exposed to COVID-19 in utero.

With ongoing endemic SARS-CoV-2 transmission, it is essential to understand how exposure to COVID-19 during pregnancy might play a role in the risk of neurodevelopmental or neurological disorders in offspring. As these conditions are usually diagnosed in preschool-aged children, robust outcome data have only recently become available.

Therefore, in the current study, we aimed to examine whether children exposed to maternal COVID-19 during pregnancy are at an increased risk of neurodevelopmental or neurological disorders. We used a national registry linkage on all children born in Norway during the pandemic (March 2020 through December 2023), when the country mandated the registration of positive polymerase chain reaction (PCR) results for SARS-CoV-2. These data also included standardized inpatient and outpatient diagnoses of relevant neurodevelopmental or neurological disorders.

## Methods

This cohort study was conducted between November 2025 and February 2026. The Regional Committee for Medical and Health Research Ethics of South/East Norway approved the study and waived the informed consent requirement for registered individuals in accordance with Norwegian legislation for use of national health registries in research. We followed the Strengthening the Reporting of Observational Studies in Epidemiology (STROBE) reporting guideline.

### Study Participants and Setting

Using a national registry linkage, we studied all children born alive in Norway between March 1, 2020, and December 31, 2023, after 22 completed gestational weeks with a birth weight greater than 500 g. Data on maternal background characteristics (Statistics Norway), birth characteristics (Medical Birth Registry of Norway), prenatal exposure to COVID-19 (Norwegian Surveillance System for Communicable Diseases), and neurodevelopmental and neurological disorders (Norwegian Patient Registry) were linked using unique national identification numbers assigned at birth.

### COVID-19 During Pregnancy

Information on laboratory-confirmed PCR positive test results for SARS-CoV-2 was obtained from mandatory reports to Norwegian Surveillance System for Communicable Diseases. Prenatal exposure to COVID-19 was defined as the mother having had a registered positive test result between conception (date of delivery minus the gestational duration in days) and the date of delivery. The main exposure of interest was any maternal COVID-19 during pregnancy. We further evaluated the risk of the outcomes of interest according to whether the last registered positive test result occurred during the first trimester (before 98 gestational days), second trimester (between 98 and 202 gestational days), or third trimester (after 202 gestational days). While the first trimester is characterized by the initial formation of brain structures and therefore is a particularly crucial stage, the second trimester is characterized by structural differentiation, and the third trimester by connection of long-range networks and substantial maturation and myelination.^26^ We also evaluated the risk according to the major circulating variants at the time the mother received a positive test result: Wild type (prior to February 1, 2021), Alpha (between February 1, 2021, and June 30, 2021), Delta (between July 1, 2021, and December 31, 2021), and Omicron (from January 1, 2022 onward). These periods were defined based on Norwegian surveillance data.^27^

In Norway, mandatory registration of all PCR tests for SARS-CoV-2 infection was implemented in the Norwegian Surveillance System for Communicable Diseases on January 31, 2020. Norway did not undertake universal testing of pregnant or delivering women for SARS-CoV-2 infection throughout the study period. Testing was predominantly conducted based on symptoms to confirm or exclude SARS-CoV-2 infection, with some additional testing conducted for specific indications, such as contact with infected persons, or mandatory testing due to travel or work. Individuals receiving a positive result on a home test, after these test kits became available, were advised to go to a testing center for a confirmatory PCR test. From mid-January 2022, regular testing was no longer recommended and testing decreased. Individuals were recommended to stay home if feeling sick, as advised for other viral diseases. Testing of patients requiring hospital care continued throughout the study period.

### Neurodevelopmental and Neurological Disorders

We evaluated a broad range of potentially relevant neurodevelopmental and neurological disorders that are captured by the Norwegian Patient Registry. These disorders included the diagnosis setting of outpatient or inpatient care and were coded according to the *International Statistical Classification of Diseases and Related Health Problems, Tenth Revision* (*ICD-10*). The 8 outcomes of interest included the following: any neurodevelopmental disorders, pervasive neurodevelopmental disorders (autism), developmental speech or language disorders, developmental motor disorders, intellectual disorders, any neurological disorders, epilepsy, and sensory impairments. The specific *ICD-10* codes used to define these outcomes are provided in eTable 1 in Supplement 1.

### Covariates

We obtained information on characteristics that might affect the associations. From the Medical Birth Registry of Norway, we obtained data on maternal age (<20, 20-24, 25-29, 30-34, 35-39, and ≥40 years), paternal age (<30, 30-39, and ≥40 years), maternal cohabitation with a partner (yes vs no), maternal parity (nulliparous vs multiparous), maternal smoking during pregnancy (yes vs no), maternal prepregnancy obesity (yes vs no; categorized as a body mass index of ≥30 [calculated as weight in kilograms divided by height in meters squared]), maternal country of birth (Norway, other European country, and rest of the world), region of delivery (South/East, West, Middle, or North Norway, a potential marker of differences in infection burden and access to services), offspring year of birth (2020, 2021, 2022, or 2023), offspring sex assigned at birth (male vs female), and preterm birth (yes vs no; <37 completed gestational weeks). Data on household income (categorized into tertiles) and maternal highest educational level obtained (≤secondary, short higher education [≤4 years], or long higher education [>4 years]) were obtained from Statistics Norway. Information on maternal history of neurodevelopmental disorders (*ICD-10* codes F7-F9) and history of epilepsy (*ICD-10* codes G40 and G41) during the 10 years prior to pregnancy with the index offspring being studied was available from the National Patient Registry, while information on prenatal exposure to maternal COVID-19 vaccination during pregnancy was available from the Norwegian Immunization Registry. Twenty-one percent of offspring studied had missing information on 1 or more covariates. We handled missing data by including a missing category. None of the observations were excluded due to missing information on covariates.

### Statistical Analysis

We show differences in the cumulative risk of the outcomes according to the exposure groups using Kaplan-Meier curves. Hazard ratios (HRs) for childhood neurodevelopmental and neurological disorders according to prenatal exposure to COVID-19 were subsequently estimated using Cox proportional hazards regression, with age in days from birth as the time metric. Each outcome was evaluated in separate models. Children were followed up from birth until the date of the diagnosis of interest, emigration, death, or August 31, 2025 (end of follow-up). Each outcome of interest was analyzed separately. Individuals were not censored if they were registered with one of the other outcomes of interest. For example, when we analyzed the risk of pervasive developmental disorders, we did not censor children if they received a diagnosis of an intellectual disorder but continued follow-up until their first diagnosis of a pervasive developmental disorder, emigration, death, or the end of follow-up.

The multivariable model adjusted for parental characteristics that could affect maternal likelihood of COVID-19 during pregnancy and offspring risk of the outcomes of interest. These characteristics included maternal age, educational level, cohabitation with a partner, parity, smoking during pregnancy, prepregnancy obesity, country of birth, history of neurodevelopmental disorder, and history of epilepsy, in addition to paternal age, region of delivery, and offspring year of birth. Preterm birth was defined as a potential mediator and therefore was not included as an adjustment variable in the main multivariable model. Offspring sex was defined as a potential effect modifier or interaction variable; therefore, we present a stratified analysis according to offspring sex. Deviations from the proportional hazard assumption were evaluated using Schoenfeld residuals. Based on this evaluation, we ran stratified Cox proportional hazards regressions by maternal educational level and country of birth, allowing the baseline hazards to vary according to these covariates. No deviations from the proportional hazards assumption were found for prenatal exposure to COVID-19. We first evaluated any prenatal exposure to COVID-19 and subsequently investigated associations according to the trimester of exposure, and the major circulating variant at the time of infection. The evaluation of the risk according to the trimester of exposure was restricted to those born at term. This approach enabled us to avoid biased associations for exposure in the third trimester, as preterm births would not have had the opportunity to be exposed during the third trimester as term births, potentially resulting in an artificially reduced risk of the outcomes among children exposed during the third trimester by including preterm birth.

We conducted several sensitivity analyses to evaluate the robustness of our findings. These analyses included one that was restricted to children born at term, one conducted only among singletons, one that excluded children exposed to maternal COVID-19 vaccination during pregnancy, and one restricted to children in utero in periods in which they could have been exposed to maternal COVID-19 throughout the pregnancy and testing was recommended (estimated start of pregnancy after March 1, 2020, and birth prior to March 1, 2022). We also conducted a sensitivity analysis using a stratified Cox proportional hazards regression analysis for gestational age to ensure that children of the same gestational age were compared.

We used a 2-sided *P* < .05 statistical significance level and therefore present 95% CIs. All analyses were conducted with Stata, version 19 (StataCorp LLC).

## Results

We studied 204 663 children (104 728 males [51.2%] and 99 935 females [48.8%]) born alive between March 1, 2020, and December 31, 2023. Mothers had a mean (SD) age of 31.6 (4.7) years, and 72.1% were born in Norway, 43.0% were nulliparous, and 61.3% had reached higher educational levels. The first case of COVID-19 was verified in Norway on February 26, 2020. Among individuals who delivered, the proportion who had received a positive test result while they were pregnant peaked between March and September 2022, with 33% or more exposed to COVID-19 during pregnancy. Of the children studied, 16 325 (8.0%) were exposed to maternal COVID-19 during pregnancy. Mothers with COVID-19 during pregnancy were older, were more likely born outside of Norway, had completed lower educational levels, had lower household income, and were more likely to have given birth in the South/East region of Norway (Table 1). The children’s mean (IQR) age at the end of follow-up was 3.6 (1.7-5.5) years. The rate of the outcomes of interest ranged from 0.6 (developmental motor disorders) to 27.8 (any neurological disorders) per 1 million follow-up days (eTable 2 in Supplement 1). The outcomes showed expected associations with male sex and preterm birth (eTable 3 in Supplement 1).

**Table 1.** Background Characteristics of Children by Exposure to Maternal COVID-19 During Pregnancy

Characteristic	Children, No. (%)		*P* value
	Without exposure to maternal COVID-19 during pregnancy (n = 188 338)	With exposure to maternal COVID-19 during pregnancy (n = 16 325)	
Maternal age, y			
<20	1557 (0.8)	94 (0.6)	<.001
20-24	18 107 (9.6)	1363 (8.3)	
25-29	62 440 (33.2)	5379 (32.9)	
30-34	71 220 (37.8)	6343 (38.9)	
35-39	29 518 (15.7)	2683 (16.4)	
≥40	5496 (2.9)	463 (2.8)	
Paternal age, y			
<30	44 391 (23.6)	3595 (22.0)	<.001
30-39	115 396 (61.3)	10 231 (62.7)	
≥40	26 198 (13.9)	2338 (14.3)	
Missing data	2353 (1.2)	161 (1.0)	
Maternal country of birth			
Norway	136 519 (72.5)	11 123 (68.1)	<.001
Other European country	24 176 (12.8)	2404 (14.7)	
Rest of the world	27 643 (14.7)	2798 (17.1)	
Region of delivery			
South/East	105 329 (55.9)	10 107 (61.9)	<.001
West	41 087 (21.8)	3244 (19.9)	
Middle	25 948 (13.8)	1868 (11.4)	
North	15 546 (8.3)	1068 (6.5)	
Missing data	428 (0.2)	38 (0.2)	
Maternal parity			
Nulliparous	82 187 (43.6)	5870 (36.0)	<.001
Multiparous	106 151 (56.4)	10 455 (64.0)	
Maternal educational level			
≤Secondary	62 975 (33.4)	5894 (36.1)	<.001
Short higher education: ≤4 y	73 304 (38.9)	6245 (38.3)	
Long higher education: >4 y	42 599 (22.6)	3413 (20.9)	
Missing data	9460 (5.0)	773 (4.7)	
Household income, tertiles			
First	62 651 (33.3)	5266 (32.3)	<.001
Second	62 291 (33.1)	5626 (34.5)	
Third	62 491 (33.2)	5426 (33.2)	
Missing data	905 (0.5)	7 (0.1)	
Maternal cohabitation with a partner			
Yes	177 758 (94.4)	15 445 (94.6)	.32
No	7613 (4.0)	646 (4.0)	
Unknown or missing data	2967 (1.6)	234 (1.4)	
Maternal history of neurodevelopmental disorder			
No	177 144 (94.1)	15 355 (94.1)	.99
Yes	11 194 (5.9)	970 (5.9)	
Maternal history of epilepsy			
No	186 629 (99.1)	16 184 (99.1)	.57
Yes	1709 (0.9)	141 (0.9)	
Maternal prepregnancy obesity			
No	150 711 (80.0)	13 125 (80.4)	.002
Yes	26 845 (14.3)	2371 (14.5)	
Missing data	10 782 (5.7)	829 (5.1)	
Maternal smoking during pregnancy			
No	162 328 (86.2)	13 984 (85.7)	<.001
Yes	3149 (1.7)	223 (1.4)	
Missing data	22 861 (12.1)	2118 (13.0)	
Prenatal exposure to COVID-19 vaccination			
No	156 037 (82.8)	10 955 (67.1)	<.001
Yes	32 301 (17.2)	5370 (32.9)	
Year of birth			
2020	44 516 (23.6)	138 (0.8)	<.001
2021	54 887 (29.1)	1445 (8.9)	
2022	37 257 (19.8)	14 425 (88.4)	
2023	51 678 (27.4)	317 (1.9)	
Preterm birth			
No	176 946 (94.0)	15 478 (94.8)	<.001
Yes	11 392 (6.0)	847 (5.2)	
Offspring sex assigned at birth			
Male	96 388 (51.2)	8340 (51.1)	.82
Female	91 950 (48.8)	7985 (48.9)	
Result of a multiple birth			
No	188 388 (97.3)	15 926 (97.6)	.03
Yes	5147 (2.7)	399 (2.4)	

The cumulative risk of the outcomes according to prenatal exposure to COVID-19 is shown in Figure. We observed no notable differences in the risk of any outcome (Table 2), with estimated adjusted HRs (AHRs) of 0.97 (95% CI, 0.88-1.08) for any neurodevelopmental disorders, 0.88 (95% CI, 0.67-1.15) for pervasive developmental disorders, 1.13 (95% CI, 0.80-1.59) for developmental speech or language disorders, 1.19 (95% CI, 0.65-2.19) for intellectual disorders, 0.83 (95% CI, 0.44-1.56) for developmental motor disorders, 0.97 (95% CI, 0.88-1.07) for any neurological disorders, 1.14 (95% CI, 0.87-1.48) for epilepsy, and 1.09; 95% CI, 0.92-1.30) for sensory impairments, with all CIs including the null value. Multivariable adjustment resulted in only modest differences in the estimated associations. The results were similar when the analysis was restricted to children born at term (eTable 4 in Supplement 1), restricted to singletons (eTable 5 in Supplement 1), or adjusted for maternal history of COVID-19 prior to pregnancy. Similarly, additional adjustment for maternal history of a broader range of psychiatric disorders, including organic mental disorders, schizophrenia, mood disorders, and neurotic disorders, did not change our findings (Table 2).

**Figure.**
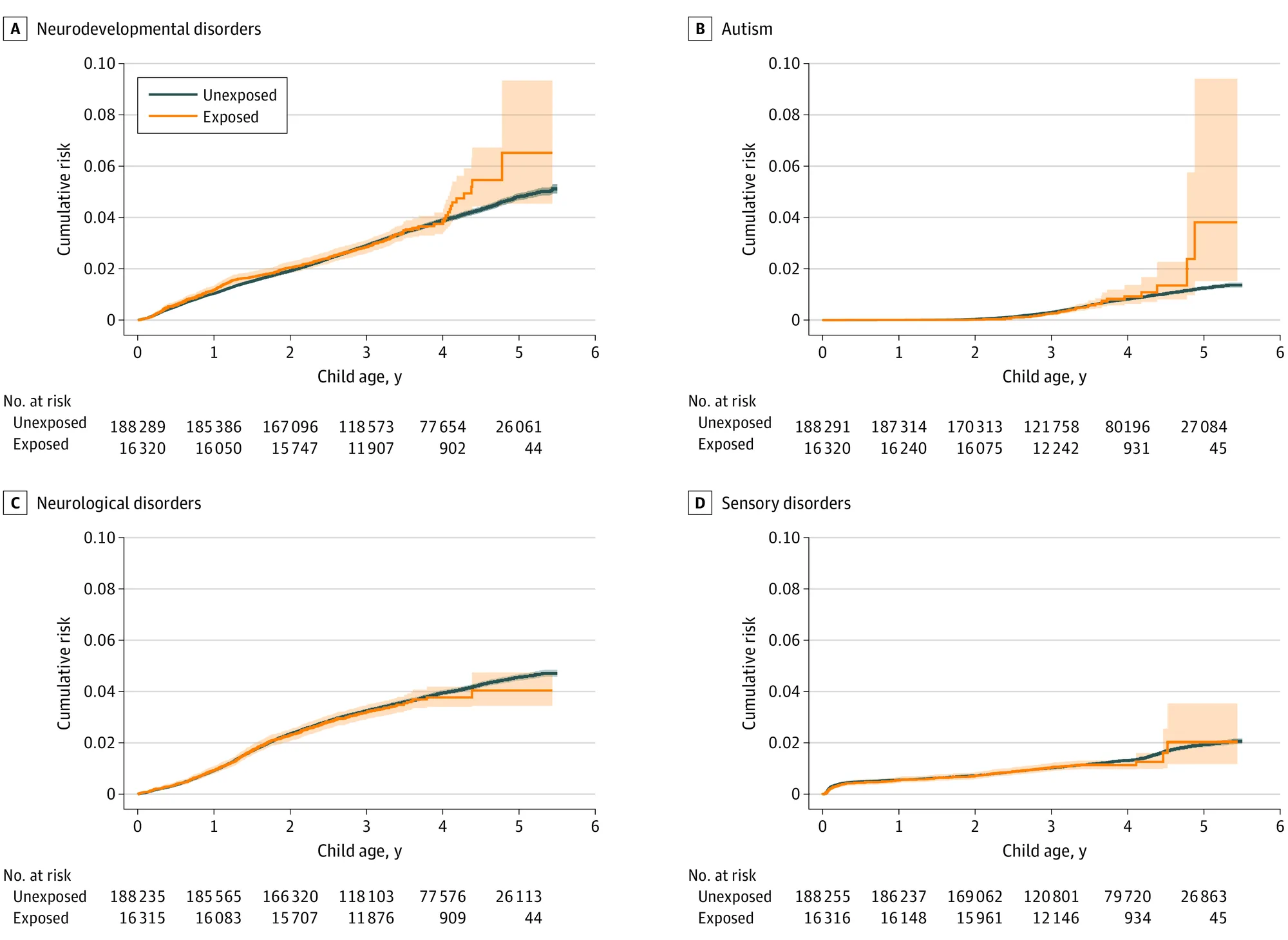
Kaplan-Meier Curves of the Risk of Neurodevelopmental and Neurological Outcomes by Prenatal Exposure to COVID-19

**Table 2.** Risk of Neurodevelopmental and Neurological Outcomes by Prenatal Exposure to COVID-19

Outcome	Children without prenatal exposure to COVID-19		Children with prenatal exposure to COVID-19		HR (95% CI)		
	Follow-up time, d	No. of cases	Follow-up time, d	No. of cases	Unadjusted	Adjusted^a^	Adjusted^b^
Any neurodevelopmental disorders	243 203 680	6523	18 965 508	518	1.01 (0.92-1.11)	0.97 (0.88-1.08)	0.97 (0.88-1.08)
Pervasive neurodevelopmental disorders (autism)	247 572 400	1223	19 286 324	70	0.98 (0.76-1.25)	0.88 (0.67-1.15)	0.88 (0.67-1.16)
Developmental speech or language disorders	247 783 600	680	19 286 528	52	1.08 (0.81-1.45)	1.13 (0.80-1.59)	1.13 (0.80-1.59)
Developmental motor disorders	248 000 672	137	19 292 920	13	1.18 (0.66-2.12)	0.83 (0.44-1.56)	0.83 (0.44-1.56)
Intellectual disorders	248 023 376	183	19 299 492	12	1.29 (0.70-2.38)	1.19 (0.65-2.19)	1.20 (0.65-2.20)
Any neurological disorders	242 821 728	6739	18 960 544	542	0.98 (0.89-1.07)	0.97 (0.88-1.07)	0.97 (0.88-1.07)
Epilepsy	247 420 560	824	19 246 388	83	1.24 (0.99-1.57)	1.14 (0.87-1.48)	1.14 (0.87-1.48)
Sensory impairments	246 011 200	2434	19 169 888	178	0.99 (0.85-1.17)	1.09 (0.92-1.30)	1.09 (0.92-1.30)

Abbreviation: HR, hazard ratio.

^a^ Adjusted for maternal age, educational level, cohabitation with a partner, parity, smoking during pregnancy, prepregnancy obesity, country of birth, history of neurodevelopmental disorder, and history of epilepsy, in addition to paternal age, region of delivery, and offspring year of birth.

^b^ Additional adjustment for maternal organic mental disorders, schizophrenia, mood disorders, and neurotic disorders.

Of the 204 663 children studied, 5514 (2.7%) were exposed to COVID-19 during the first trimester, 6164 (3.0%) during the second trimester, and 4647 (2.3%) during the third trimester. In line with the main results, we observed no robust evidence for an increased risk of any outcome of interest associated with exposure to COVID-19 during specific trimesters (Table 3). Overall, 619 children (0.3%) were exposed to the Wild-type variant, 605 (0.3%) to the Alpha variant, 2059 (1.0%) to the Delta variant, and 13 042 (6.4%) to the Omicron variant of the virus. There were no differences in the risk of the outcomes according to the dominant circulating variant at the time the pregnant mother was infected with COVID-19 (eTable 6 in Supplement 1). Sensitivity analysis that excluded children who were exposed to maternal COVID-19 vaccination during pregnancy (n = 37 671) yielded similar results (eTable 7 in Supplement 1).

**Table 3.** Risk of Neurodevelopmental and Neurological Outcomes by Trimester of Prenatal Exposure to COVID-19 Restricted to Children Born at Term

Outcome and exposure group	Follow-up time, d	No. of cases^a^	HR (95% CI)	
			Unadjusted	Adjusted^b^
Any neurodevelopmental disorders				
Unexposed	229 185 760	5633	1 [Reference]	1 [Reference]
Exposed in first trimester	5 619 923	129	0.95 (0.79-1.13)	0.90 (0.75-1.09)
Exposed in second trimester	6 729 154	188	1.14 (0.98-1.33)	1.09 (0.93-1.27)
Exposed in third trimester	5 475 473	140	1.04 (0.87-1.23)	1.01 (0.85-1.21)
Pervasive neurodevelopmental disorders (autism)				
Unexposed	232 917	1100	1 [Reference]	1 [Reference]
Exposed in first trimester	5 695 247	16	0.99 (0.60-1.64)	0.90 (0.54-1.52)
Exposed in second trimester	6 849 205	23	0.94 (0.61-1.44)	0.82 (0.53-1.27)
Exposed in third trimester	5 562 474	27	1.14 (0.77-1.69)	1.03 (0.69-1.55)
Any neurological disorders				
Unexposed	228 654 576	6063	1 [Reference]	1 [Reference]
Exposed in first trimester	5 605 932	159	0.99 (0.85-1.17)	1.01 (0.85-1.20)
Exposed in second trimester	6 737 794	180	0.96 (0.82-1.11)	0.97 (0.83-1.14)
Exposed in third trimester	5 466 975	151	1.01 (0.86-1.19)	1.01 (0.86-1.20)
Epilepsy				
Unexposed	232 792 800	726	1 [Reference]	1 [Reference]
Exposed in first trimester	5 684 544	22	1.17 (0.76-1.80)	1.08 (0.68-1.72)
Exposed in second trimester	6 834 832	32	1.44 (1.01-2.07)	1.33 (0.90-1.97)
Exposed in third trimester	5 552 823	20	1.13 (0.72-1.79)	1.05 (0.66-1.67)
Sensory impairments				
Unexposed	231 538 400	2182	1 [Reference]	1 [Reference]
Exposed in first trimester	5 665 849	54	1.07 (0.81-1.42)	1.19 (0.89-1.61)
Exposed in second trimester	6 811 564	56	0.93 (0.71-1.22)	1.03 (0.78-1.37)
Exposed in third trimester	5 534 777	43	0.88 (0.65-1.20)	0.95 (0.69-1.31)

Abbreviation: HR, hazard ratio.

^a^ This analysis excluded 164 children exposed to maternal COVID-19 during multiple trimesters.

^b^ Adjusted for maternal age, educational level, cohabitation with a partner, parity, smoking during pregnancy, prepregnancy obesity, country of birth, history of neurodevelopmental disorder, and history of epilepsy, in addition to paternal age, region of delivery, and offspring year of birth.

A Cox proportional hazards regression analysis stratified by gestational age did not alter the findings. There were also no notable differences in the associations according to offspring sex, with all sex-specific CIs overlapping and all interactions terms having *P* > .20 (eFigure 1 in Supplement 1). We observed an increased risk of developmental speech or language disorders in the analyses restricted to children conceived after March 1, 2020, and born prior to March 1, 2022 (AHR, 2.37; 95% CI, 1.52-3.71) (eTable 8 in Supplement 1), who were able to be registered as exposed throughout the entire duration of pregnancy. When we examined these results by excluding children conceived prior to March 1, 2020, and children born after March 1, 2022, separately, we found that the difference in the results from the main analyses was associated with the exclusion of those born after March 1, 2022, when COVID-19 testing was no longer routinely recommended in the presence of symptoms (Table 4).

**Table 4.** Risk of Neurodevelopmental and Neurological Outcomes by Prenatal Exposure to COVID-19 Excluding Children Conceived Before March 1, 2020, and Children Born After March 1, 2022

Restriction and outcome	Children without prenatal exposure to COVID-19		Children with prenatal exposure to COVID-19		HR (95% CI)	
	Follow-up time, d	No. of cases	Follow-up time, d	No. of cases	Unadjusted	Adjusted^a^
Excluding children conceived before March 1, 2020						
Any neurodevelopmental disorders	168 934 160	4503	18 769 440	510	1.01 (0.92-1.12)	0.97 (0.88-1.08)
Pervasive neurodevelopmental disorders (autism)	171 673 472	699	19 086 264	67	0.96 (0.74-1.24)	0.88 (0.67-1.16)
Developmental speech or language disorder	171 774 400	399	19 086 872	51	1.13 (0.83-1.53)	1.12 (0.79-1.59)
Developmental motor disorder	171 852 096	100	19 092 546	13	1.18 (0.66-2.13)	0.83 (0.44-1.57)
Any neurological disorders	168 689 152	4801	18 766 908	536	0.98 (0.90-1.08)	0.97 (0.87-1.07)
Epilepsy	171 493 792	592	19 047 200	81	1.23 (0.97-1.56)	1.12 (0.85-1.46)
Sensory impairments	170 654 352	1596	18 972 226	175	1.00 (0.85-1.18)	1.09 (0.91-1.30)
Excluding children born after March 1, 2022						
Any neurodevelopmental disorders	170 047 280	4616	3 998 927	137	1.25 (1.05-1.49)	1.10 (0.92-1.33)
Pervasive neurodevelopmental disorders (autism)	173 488 912	1095	4 079 080	32	1.45 (1.01-2.08)	0.96 (0.66-1.40)
Developmental speech or language disorder	173 698 704	546	4 079 287	25	2.17 (1.44-3.27)	2.22 (1.44-3.41)
Developmental motor disorder	173 917 600	85	4 088 024	<5	NA	NA
Any neurological disorders	169 743 632	4550	4 009 347	105	0.93 (0.76-1.13)	0.95 (0.78-1.17)
Epilepsy	173 462 944	549	4 071 880	20	1.49 (0.94-2.35)	1.37 (0.85-2.21)
Sensory impairments	172 308 192	1792	4 060 444	31	0.78 (0.53-1.14)	0.99 (0.67-1.45)

Abbreviations: HR, hazard ratio; NA, not applicable.

^a^ Adjusted for maternal age, educational level, cohabitation with a partner, parity, smoking during pregnancy, prepregnancy obesity, country of birth, history of neurodevelopmental disorder, and history of epilepsy, in addition to paternal age, region of delivery, and offspring year of birth.

In the analysis excluding children born after March 1, 2022, children exposed to maternal COVID-19 during pregnancy had an increased risk of developmental speech or language disorders (AHR, 2.22; 95% CI, 1.44-3.41). We observed that those excluded because they were conceived prior to March 1, 2020, were exposed later in the pregnancy, while the gestational timing of exposure among those excluded because they were born after March 1, 2022, was balanced throughout the pregnancy with a higher number exposed early in the pregnancy (eFigure 2 in Supplement 1).

## Discussion

In this large population-based cohort study, we did not find evidence that children born to mothers with COVID-19 while pregnant have an increased risk of neurodevelopmental or neurological disorders. Our findings were robust across a range of sensitivity analyses and did not vary with trimester of infection or dominant-circulating variants of the SARS-CoV-2 virus at the time of maternal infection. The only exception was developmental speech or language disorders, for which our sensitivity analysis excluding children born after March 1, 2022, when testing for SARS-CoV-2 infection was no longer recommended, showed a 2-fold increased risk among children exposed to maternal COVID-19 during pregnancy.

Our results only partly support findings from 3 related studies based on similar administrative data from Massachusetts health systems in the US, suggesting an increased risk of neurodevelopmental disorders among children exposed to maternal prenatal SARS-CoV-2 infection.^22,23,24^ Of these 3 studies, the first evaluated neurodevelopmental outcomes at 1 year of age^24^ in approximately 7000 children (222 exposed to COVID-19 during pregnancy); it reported an increased risk of neurodevelopmental outcomes among children of mothers with SARS-CoV-2 infection during pregnancy (AOR, 1.86; 95% CI, 1.03-3.36). In the second study, the same group further investigated sex differences and evaluated outcomes up to 18 months of age among 18 555 children (883 exposed to COVID-19 during pregnancy).^23^ Based on their observations, the authors proposed that the association was primarily observed among male offspring (AOR, 1.94; 95% CI, 1.12-3.17) and not female offspring (AOR, 0.89; 95% CI, 0.39-1.76). However, there was a high uncertainty in these results, with overlapping sex-specific CIs, highlighting the need for cautious interpretation of these stratified results. In the third study, the authors examined, outcomes up to 3 years of age and replicated their previous findings that indicated an increased risk (AOR, 1.29; 95% CI, 1.05-1.57).^22^ None of these studies looked at developmental speech and language disorders specifically, but these disorders were grouped together with other neurodevelopmental diagnoses. A more recent study suggest that an increased risk of autism among children exposed to COVID-19 during pregnancy was only observed among female offspring (AHR, 1.55; 95% CI, 1.05-1.97), highlighting the uncertainty in the findings of existing studies.^25^ That study found no indication of an increased risk of developmental speech or language disorders (AHR, 1.01; 95% CI, 0.91-1.12).^25^

Our study indicates that children exposed to maternal SARS-CoV-2 infection may have an increased risk of developmental speech or language delays. This observation was supported by our sensitivity analysis excluding children born after March 1, 2022, where the risk of exposure misclassification was minimized. We are therefore unable to exclude the possibility of an association between maternal infection in this group and offspring outcomes. This finding could be explained by the implications of SARS-CoV-2 infection for the function of placental Hofbauer cells, which has been found to reflect the outcome of prenatal exposures in fetal brain microglia, immune cells important to neurodevelopment.^28,29^ Other plausible explanations include implications of the SARS-CoV-2 virus for fetoplacental complement pathways, leading to inflammation and compromised blood vessel integrity in the fetal brain.^8,30,31^ However, we may have expected a more consistent pattern for the different outcomes evaluated if these biological mechanisms were playing a role. An alternative explanation could be associated with pandemic mitigation measures (lock-down restrictions), which limited early socialization and stimulation of language development.^32,33,34^ Our finding specifically for developmental speech or language disorders should be further examined in future studies.

### Strengths and Limitations 

Strengths of our study include the large sample size consisting of all children born in Norway during a specific period and our ability to adjust for maternal conditions that, to some degree, could confound associations with an underlying genetic predisposition to these disorders. This sample size also enabled us to study whether the risk varied according to SARS-CoV-2 variants and timing of exposure, which, to our knowledge, had not been previously studied with adequate statistical power.

We also acknowledge the study limitations. We followed up the children for a mean (IQR) duration of 3.6 (1.7-5.5) years. A longer follow-up might have enabled us to identify those whose neurodevelopmental diagnoses occurred later in childhood, although the majority of severe neurodevelopmental disorders are diagnosed within this age range.^35^ We captured only positive PCR test results. Asymptomatic or mild cases of COVID-19 were therefore unlikely to have been captured, which could have resulted in a misclassification of the exposure. The testing recommendations in Norway also changed during the study period, and there was no universal recommendation for PCR testing at the presence of symptoms after March 2022. This situation could have resulted in an underestimation of prenatal exposure to COVID-19 and therefore a potential attenuation of the associations of interest. In particular, we might have underestimated the associations of interest given that children born toward the end of the study period were less likely to have been classified as exposed to maternal COVID-19 during pregnancy. This underestimation seemed to be supported by our sensitivity analyses restricted to children with an estimated start of pregnancy after March 1, 2020, and who were born prior to March 1, 2022, who all had a similar likelihood of being captured as the exposed group based on testing strategies throughout the pregnancy; among these children, we observed an increased risk of developmental speech or language disorders.

We did not observe an increased risk of pervasive developmental disorders or epilepsy, to be considered as more severe outcomes, among these offspring, in line with our main analysis. The database on the positive PCR test results for SARS-CoV-2 infection also did not include information on the variant of the virus, and we relied on the dominant-circulating variants as a proxy for infection with different variants. The majority of exposed children were exposed to maternal infection with the Omicron variant, and the study may have been underpowered to detect associations with other variants. We observed a tendency for an increased risk of any neurodevelopmental disorder among offspring born to mothers infected with the Delta variant, which was attenuated after multivariable adjustment. This finding most likely reflects confounding, which was accounted for. Maternal SARS-CoV-2 infection increases the risk of fetal death (both miscarriage and stillbirth).^11,14^ Our restriction to live births could therefore have resulted in a selection bias if pregnancy resulting in a fetal death would have had an increased risk of neurodevelopmental and neurological outcomes if it had survived. We also did not have information on maternal use of any medications to relieve their symptoms of COVID-19, which could act as potential mediators of the associations of interest, as maternal paracetamol use has been reported to be associated with neurodevelopmental disorders.^36^ We did not have any information on the clinical severity for the positive cases of COVID-19. Additionally, there were too few children born to mothers hospitalized for COVID-19 for any meaningful analyses to be conducted. We were therefore unable to comment on whether exposure to more severe COVID-19 during pregnancy might change the risk of neurodevelopmental or neurological disorders among offspring.

## Conclusions

In this population-based cohort study, children exposed to maternal COVID-19 prenatally did not show an increased risk of neurodevelopmental disorders, except for an increased risk of speech or language disorders among those exposed in the early phase of the pandemic. These findings warrant replication in other populations and settings.

## Notes

Supplement 1. **eTable 1.** ICD-10 Codes Used to Define the Neurodevelopmental and Neurological Outcomes of Interest **eTable 2.** Incidence Rate and Age at First Registration of the Neurodevelopmental and Neurological Diagnoses of Interest **eTable 3.** Risk of the Neurodevelopmental and Neurological Diagnoses of Interest According to Male Sex and Preterm Birth **eTable 4.** Risk of Neurodevelopmental and Neurological Outcomes According to Prenatal Exposure to COVID-19 Infection Restricted to Children Born at Term **eTable 5.** Risk of Neurodevelopmental and Neurological Outcomes According to Prenatal Exposure to COVID-19 Infection Restricted to Singletons **eTable 6.** Risk of Neurodevelopmental and Neurological Outcomes According to Prenatal Exposure to COVID-19 Infection by Major Circulating Variant at the Time of Infection **eTable 7.** Risk of Neurodevelopmental and Neurological Outcomes According to Prenatal Exposure to COVID-19 Infection Restricted to Children Not Exposed to Maternal Vaccination Against Covid-19 During Pregnancy **eTable 8.** Risk of Neurodevelopmental and Neurological Outcomes According to Prenatal Exposure to COVID-19 Infection Restricted to Children Able to be Exposed Throughout the Entire Duration of the Pregnancy (Estimated Start of Pregnancy From March 1st 2020 and Birth Prior to March 1st 2022) **eFigure 1.** Risk of Neurodevelopmental and Neurological Outcomes According to Prenatal Exposure to COVID-19 Infection Stratified by Sex **eFigure 2.** The Gestational Day of the Positive Test Among Children Conceived Prior to March 1st 2020 and Delivered After March 1st 2022

Supplement 2.
Data Sharing Statement
