# Error in the Figure Title

**DOI:** 10.1001/jamanetworkopen.2026.42564

**Published:** 2026-10-09

In the Original Investigation titled, “Prenatal COVID-19 Exposure and Neurodevelopmental and Neurological Disorders in Children”^1^ published online September 3, 2026, the Figure title incorrectly indicated that the exposure was vaccination. In fact, the exposure was infection. This article has been corrected.^1^
