# Supplementary material for: Prenatal COVID-19 Exposure and Neurodevelopmental and Neurological Disorders in Children
Source: JAMA Netw Open. 2026 Sep 3;9(9):e2631847. doi: 10.1001/jamanetworkopen.2026.31847 (PMC13543023; doi:10.1001/jamanetworkopen.2026.31847)
Supplement: Supplementary file 1 [file jamanetwopen-e2631847-s001.pdf]

Supplemental Online Content

Magnus MC, Eide HN, Burgner DP, Stephansson O, Eldevik Håberg S. Prenatal COVID-19 exposure and neurodevelopmental and neurological disorders in children. *JAMA Netw Open*. 2026;9(9): e2631847. doi:10.1001/jamanetworkopen.2026.31847

eTable 1. ICD-10 Codes Used to Define the Neurodevelopmental and Neurological Outcomes of Interest

eTable 2. Incidence Rate and Age at First Registration of the Neurodevelopmental and Neurological Diagnoses of Interest

eTable 3. Risk of the Neurodevelopmental and Neurological Diagnoses of Interest According to Male Sex and Preterm Birth

eTable 4. Risk of Neurodevelopmental and Neurological Outcomes According to Prenatal Exposure to COVID-19 Infection Restricted to Children Born at Term

eTable 5. Risk of Neurodevelopmental and Neurological Outcomes According to Prenatal Exposure to COVID-19 Infection Restricted to Singletons

eTable 6. Risk of Neurodevelopmental and Neurological Outcomes According to Prenatal Exposure to COVID-19 Infection by Major Circulating Variant at the Time of Infection

eTable 7. Risk of Neurodevelopmental and Neurological Outcomes According to Prenatal Exposure to COVID-19 Infection Restricted to Children Not Exposed to Maternal Vaccination Against Covid-19 During Pregnancy

eTable 8. Risk of Neurodevelopmental and Neurological Outcomes According to Prenatal Exposure to COVID-19 Infection Restricted to Children Able to be Exposed Throughout the Entire Duration of the Pregnancy (Estimated Start of Pregnancy From March 1st 2020 and Birth Prior to March 1st 2022)

eFigure 1. Risk of Neurodevelopmental and Neurological Outcomes According to Prenatal Exposure to COVID-19 Infection Stratified by Sex

eFigure 2. The Gestational Day of the Positive Test Among Children Conceived Prior to March 1st 2020 and Delivered After March 1st 2022

This supplemental material has been provided by the authors to give readers additional information about their work.

© 2026 Magnus MC et al. *JAMA Network Open*.

eTable 1 ICD-10 codes used to define the neurodevelopmental and neurological outcomes of interest

Outcome	Contact with specialist healthcare services (ICD-10)
**Any neurodevelopmental diagnoses**	F70–F79; F80; F82; F83; F84.0; F84.1; F84.3; F84.5; F84.8; F84.9; F88; F89; F90; F95; F98; R62.0
Pervasive developmental disorders (autism)	F840; F841; F843; F845; F848; F849
Developmental speech or language disorder	F80
Developmental motor disorder	F82
Intellectual disorders	F70–F79; F83
**Any neurological diagnoses**	G40–G47; G80–G83
Epilepsy	G40; G41;
**Sensory impairments**	H90; H91; H54

© 2026 Magnus MC et al. *JAMA Network Open*.

eTable 2 Incidence rate and age at first registration of the neurodevelopmental and neurological diagnoses of interest

**Outcome**	**Risk per 1,000,000 follow-up days**	**Age at first registration Mean (IQR)**
**Any neurodevelopmental diagnoses**	26,9	1.75 (0.80, 2.88)
Pervasive developmental disorders (autism)	4,8	3.24 (2.68, 3.80)
Developmental speech or language disorder	2,7	2.86 (2.31, 3.60)
Developmental motor disorder	0,6	1.34 (0.88, 1.85)
Intellectual disorders	0,7	3.34 (2.38, 4.08)
**Any neurological diagnoses**	27,8	1.56 (0.96, 2.41)
Epilepsy	3,4	1.36 (0.57, 2.46)
**Sensory impairments**	9,8	1.47 (0.13, 3.01)

© 2026 Magnus MC et al. *JAMA Network Open*.

eTable 3 Risk of the neurodevelopmental and neurological diagnoses of interest according to male sex and preterm birth

Outcome	Male sex<br>Unadjusted Hazard Ratio (95% CI)	Preterm birth<br>Unadjusted Hazard Ratio (95% CI)
**Any neurodevelopmental diagnoses**	1.55 (1.47 to 1.63)	2.55 (2.37 to 2.75)
Pervasive developmental disorders (autism)	2.73 (2.40 to 3.11)	1.75 (1.44 to 2.14)
Developmental speech or language disorder	3.12 (2.62 to 3.71)	1.23 (0.92 to 1.64)
Developmental motor disorder	1.09 (0.79 to 1.51)	2.46 (1.52 to 3.98)
Intellectual disorders	1.60 (1.19 to 2.15)	2.83 (1.89 to 4.24)
**Any neurological diagnoses**	1.19 (1.14 to 1.25)	1.79 (1.66 to 1.94)
Epilepsy	0.95 (0.83 to 1.09)	2.15 (1.74 to 2.64)
**Sensory impairments**	1.43 (1.32 to 1.55)	1.92 (1.68 to 2.19)

© 2026 Magnus MC et al. *JAMA Network Open*.

eTable 4 Risk of neurodevelopmental and neurological outcomes according to prenatal exposure to COVID-19 infection restricted to children born at term

Outcome	Unexposed		Exposed		Unadjusted Hazard Ratio (95% CI)	Adjusted Hazard Ratio (95% CI)
	Follow-up time in days	Number of cases	Follow-up time in days	Number of cases		
**Any neurodevelopmental diagnoses**	229185760	5633	18006892	465	1.06 (0.96 to 1.16)	1.01 (0.91 to 1.13)
Pervasive developmental disorders (autism)	232917808	1100	18292656	67	1.03 (0.80 to 1.34)	0.92 (0.70 to 1.22)
Developmental speech or language disorder	233100048	631	18293592	49	1.10 (0.81 to 1.49)	1.15 (0.81 to 1.64)
Developmental motor disorder	233310096	118	18299308	12	1.27 (0.69 to 2.33)	0.92 (0.47 to 1.80)
Intellectual disorders	233331840	155	18305884	11	1.47 (0.77 to 2.82)	1.42 (0.76 to 2.67)
**Any neurological diagnoses**	228654576	6063	17994400	494	0.98 (0.89 to 1.08)	0.99 (0.90 to 1.10)
Epilepsy	232792800	726	18258192	74	1.25 (0.98 to 1.60)	1.15 (0.87 to 1.53)
**Sensory impairments**	231538400	2182	18198182	153	0.95 (0.80 to 1.12)	1.04 (0.87 to 1.26)

Adjusted for maternal age, educational level, living with a partner, parity, smoking during pregnancy, obesity, country of birth, history of neurodevelopmental disorder, and history of epilepsy, in addition to paternal age, region of delivery and offspring year of birth.

© 2026 Magnus MC et al. *JAMA Network Open*.

eTable 5 Risk of neurodevelopmental and neurological outcomes according to prenatal exposure to COVID-19 infection restricted to singletons

Outcome	Unexposed		Exposed		Unadjusted Hazard Ratio (95% CI)	Adjusted Hazard Ratio (95% CI)
	Follow-up time in days	Number of cases	Follow-up time in days	Number of cases		
**Any neurodevelopmental diagnoses**	236738704	6235	18507150	497	1.01 (0.92 to 1.11)	0.97 (0.87 to 1.07)
Pervasive developmental disorders (autism)	240915728	1168	18816198	70	1.02 (0.79 to 1.31)	0.91 (0.70 to 1.20)
Developmental speech or language disorder	241108512	673	18817376	50	1.05 (0.78 to 1.42)	1.08 (0.76 to 1.53)
Developmental motor disorder	241323248	135	18822792	13	1.20 (0.67 to 2.15)	0.82 (0.44 to 1.54)
Intellectual disorders	241348288	176	18829366	12	1.34 (0.73 to 2.49)	1.21 (0.66 to 2.23)
**Any neurological diagnoses**	236314272	6511	18497750	524	0.98 (0.89 to 1.07)	0.97 (0.88 to 1.07)
Epilepsy	240760080	799	18778716	78	1.20 (0.94 to 1.52)	1.11 (0.85 to 1.47)
**Sensory impairments**	239401616	2358	18709200	164	0.94 (0.80 to 1.11)	1.03 (0.86 to 1.24)

Adjusted for maternal age, educational level, living with a partner, parity, smoking during pregnancy, obesity, country of birth, history of neurodevelopmental disorder, and history of epilepsy, in addition to paternal age, region of delivery and offspring year of birth.

© 2026 Magnus MC et al. *JAMA Network Open*.

eTable 6 Risk of neurodevelopmental and neurological outcomes according to prenatal exposure to COVID-19 infection by major circulating variant at the time of infection

Outcome	Exposure group	Follow-up time in days	Number of cases	Unadjusted hazard ratio (95% CI)	Adjusted hazard ratio (95% CI)
**Any neurodevelopmental diagnoses**	Unexposed	243203680	6523	Reference	Reference
	Wild type	977113	29	1.11 (0.76 to 1.60)	0.88 (0.60 to 1.27)
	Alpha	859742	35	1.51 (1.08 to 2.11)	1.17 (0.83 to 1.65)
	Delta	2499674	89	1.32 (1.06 to 1.63)	1.16 (0.93 to 1.45)
	Omicron	14628980	365	0.93 (0.83 to 1.03)	0.93 (0.82 to 1.05)
Pervasive developmental disorders (autism)	Unexposed	247572400	1223	Reference	Reference
	Wild type	990623	11	1.80 (0.98 to 3.30)	1.01 (0.55 to 1.86)
	Alpha	880017	9	1.82 (0.93 to 3.56)	0.98 (0.50 to 1.92)
	Delta	2552012	16	1.48 (0.89 to 2.45)	1.08 (0.64 to 1.81)
	Omicron	14863673	34	0.68 (0.48 to 0.96)	0.75 (0.51 to 1.09)
**Any neurological diagnoses**	Unexposed	242821728	6739	Reference	Reference
	Wild type	974046	21	0.81 (0.52 to 1.26)	0.81 (0.53 to 1.26)
	Alpha	867620	19	0.80 (0.50 to 1.26)	0.82 (0.51 to 1.29)
	Delta	2506527	80	1.11 (0.88 to 1.39)	1.12 (0.89 to 1.41)
	Omicron	14612352	422	0.98 (0.88 to 1.08)	0.97 (0.87 to 1.09)
Epilepsy	Unexposed	247420560	824	Reference	Reference
	Wild type	989975	6	1.89 (0.83 to 4.27)	1.94 (0.86 to 4.41)
	Alpha	879205	5	1.73 (0.71 to 4.23)	1.80 (0.73 to 4.44)
	Delta	2548069	9	1.03 (0.53 to 2.02)	0.94 (0.48 to 1.85)
	Omicron	14829140	63	1.21 (0.93 to 1.57)	1.07 (0.79 to 1.44)
**Sensory impairments**	Unexposed	246011200	2434	Reference	Reference
	Wild type	985489	11	1.13 (0.62 to 2.06)	1.25 (0.69 to 2.30)
	Alpha	879090	6	0.74 (0.29 to 1.89)	0.86 (0.33 to 2.21)
	Delta	2539232	20	0.85 (0.55 to 1.34)	0.97 (0.62 to 1.53)

© 2026 Magnus MC et al. *JAMA Network Open*.

	Omicron	14766078	141	1.02 (0.86 to 1.22)	1.11 (0.91 to 1.36)

Adjusted for maternal age, educational level, living with a partner, parity, smoking during pregnancy, obesity, country of birth, history of neurodevelopmental disorder, and history of epilepsy, in addition to paternal age, region of delivery and offspring year of birth.

© 2026 Magnus MC et al. *JAMA Network Open*.

eTable 7 Risk of neurodevelopmental and neurological outcomes according to prenatal exposure to COVID-19 infection restricted to children not exposed to maternal vaccination against Covid-19 during pregnancy

Outcome	Unexposed		Exposed		Unadjusted Hazard Ratio (95% CI)	Adjusted Hazard Ratio (95% CI)
	Follow-up time in days	Number of cases	Follow-up time in days	Number of cases		
**Any neurodevelopmental diagnoses**	205213152	5627	12563975	375	1.08 (0.97 to 1.21)	1.02 (0.90 to 1.16)
Pervasive developmental disorders (autism)	209010000	1113	12787108	58	1.19 (0.90 to 1.56)	1.03 (0.77 to 1.38)
Developmental speech or language disorder	209209472	611	12791226	35	1.04 (0.73 to 1.49)	1.05 (0.68 to 1.62)
Developmental motor disorder	209424576	113	12794081	9	1.26 (0.63 to 2.52)	0.80 (0.37 to 1.72)
Intellectual disorders	209432224	168	12798621	9	1.36 (0.68 to 2.75)	1.11 (0.56 to 2.20)
**Any neurological diagnoses**	204950064	5624	12586437	345	0.94 (0.84 to 1.05)	0.92 (0.81 to 1.05)
Epilepsy	208925888	693	12769673	47	1.06 (0.78 to 1.43)	0.94 (0.66 to 1.34)
**Sensory impairments**	207686400	2085	12708817	126	1.05 (0.87 to 1.27)	1.22 (0.99 to 1.52)

Adjusted for maternal age, educational level, living with a partner, parity, smoking during pregnancy, obesity, country of birth, history of neurodevelopmental disorder, and history of epilepsy, in addition to paternal age, region of delivery and offspring year of birth.

© 2026 Magnus MC et al. *JAMA Network Open*.

eTable 8 Risk of neurodevelopmental and neurological outcomes according to prenatal exposure to COVID-19 infection restricted to children able to be exposed throughout the entire duration of the pregnancy (estimated start of pregnancy from March 1st 2020 and birth prior to March 1st 2022)

Outcome	Unexposed		Exposed		Unadjusted Hazard Ratio (95% CI)	Adjusted Hazard Ratio (95% CI)
	Follow-up time in days	Number of cases	Follow-up time in days	Number of cases		
**Any neurodevelopmental diagnoses**	95777768	2596	3802858	129	1.24 (1.03 to 1.49)	1.12 (0.92 to 1.35)
Pervasive developmental disorders (autism)	97589992	571	3879020	29	1.41 (0.96 to 2.06)	0.97 (0.65 to 1.45)
Developmental speech or language disorder	97689488	265	3879632	24	2.45 (1.60 to 3.76)	2.37 (1.52 to 3.71)
**Any neurological diagnoses**	95611056	2612	3815709	99	0.92 (0.75 to 1.13)	0.94 (0.76 to 1.16)
Epilepsy	97536176	317	3872691	18	1.41 (0.87 to 2.29)	1.28 (0.77 to 2.11)
**Sensory impairments**	96951344	954	3862781	28	0.75 (0.50 to 1.13)	0.94 (0.62 to 1.42)

Adjusted for maternal age, educational level, living with a partner, parity, smoking during pregnancy, obesity, country of birth, history of neurodevelopmental disorder, and history of epilepsy, in addition to paternal age, region of delivery and offspring year of birth.

© 2026 Magnus MC et al. *JAMA Network Open*.

eFigure 1 Risk of neurodevelopmental and neurological outcomes according to prenatal exposure to Covid-19 infection stratified by sex

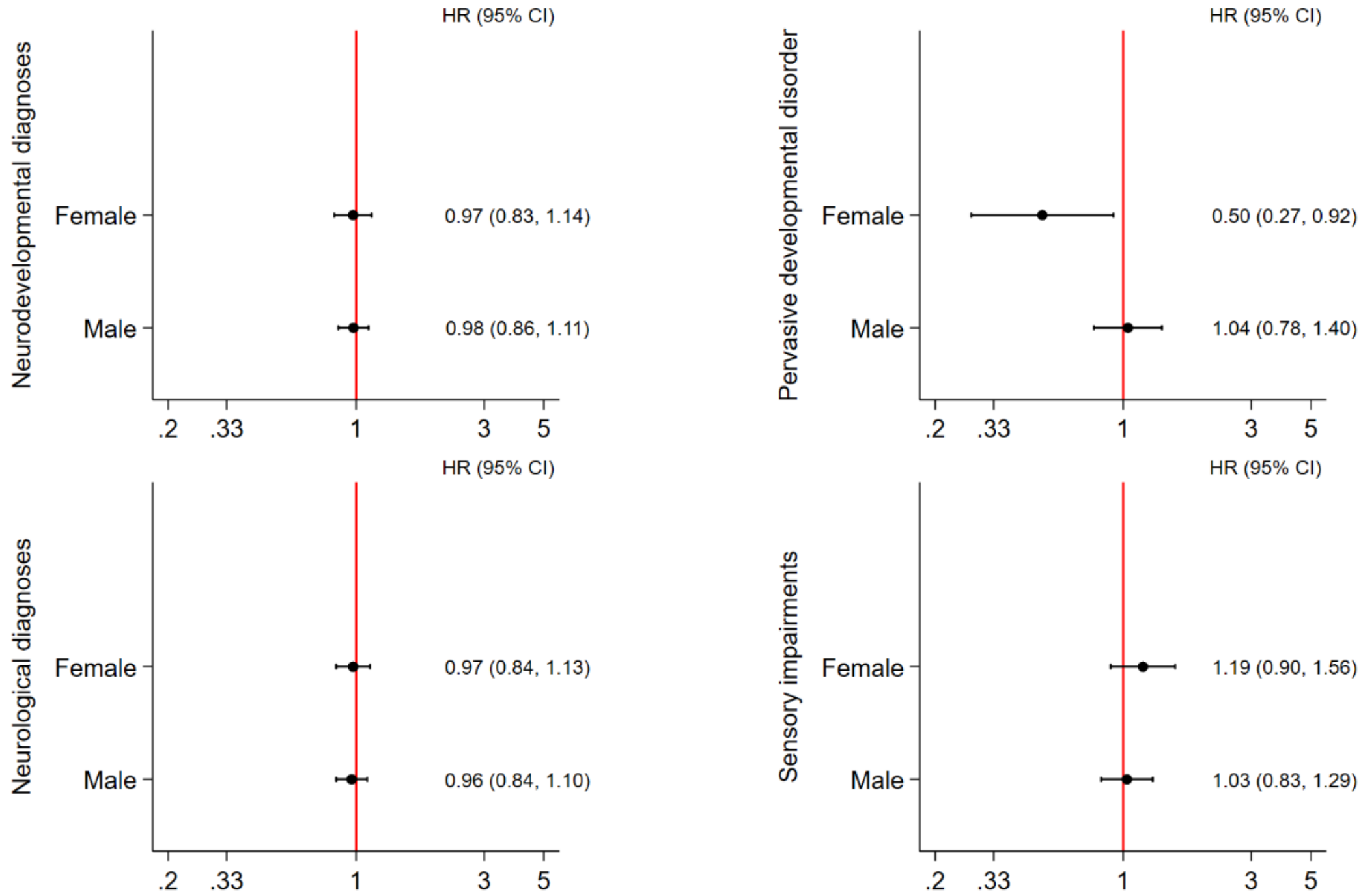

Adjusted for maternal age, educational level, living with a partner, parity, smoking during pregnancy, obesity, country of birth, history of neurodevelopmental disorder, and history of epilepsy, in addition to paternal age, region of delivery and offspring year of birth.

© 2026 Magnus MC et al. *JAMA Network Open*.

eFigure 2 The gestational day of the positive test among children conceived prior to March 1st 2020 and delivered after March 1st 2022

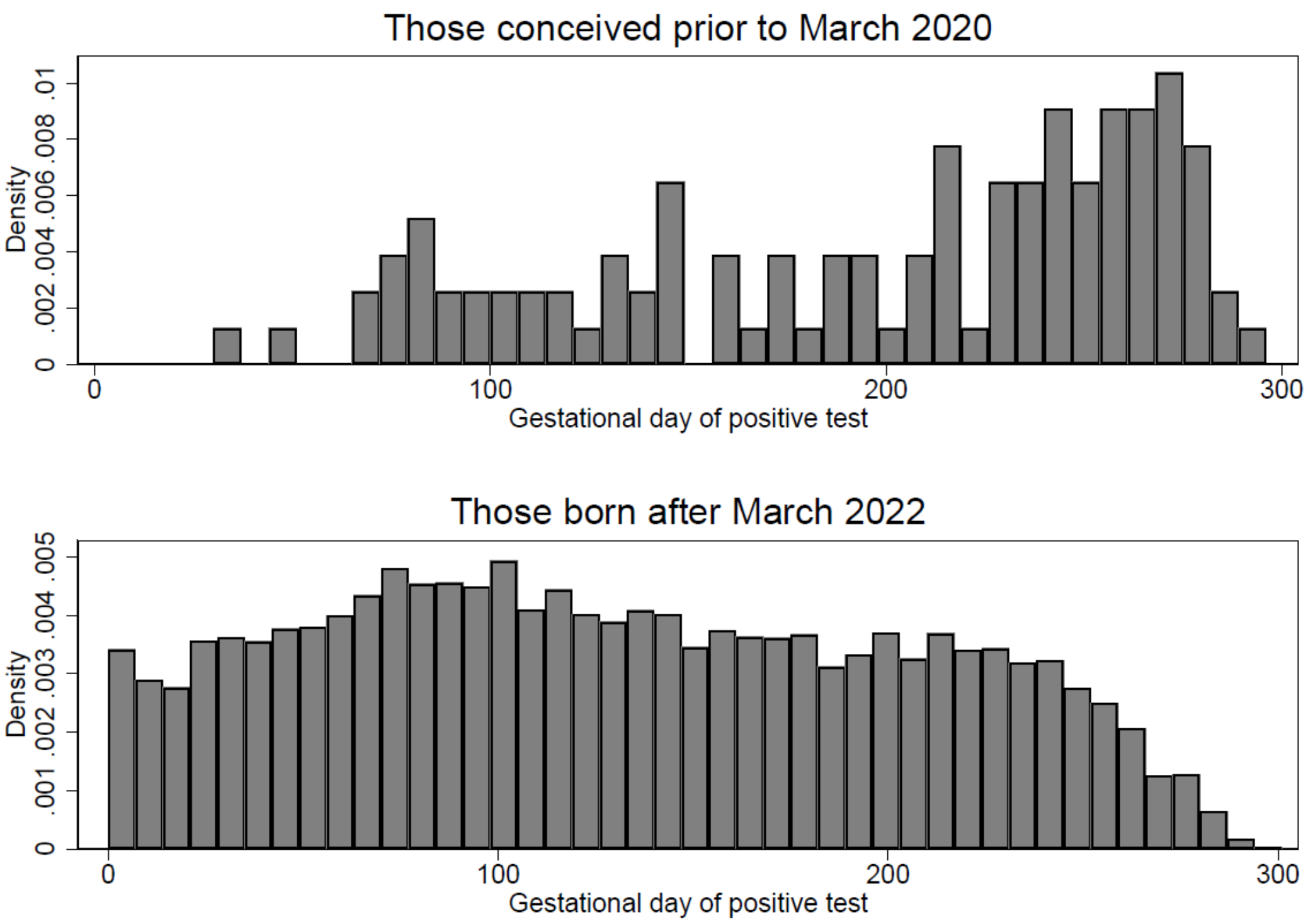

© 2026 Magnus MC et al. *JAMA Network Open*.
