# Supplementary material for: Prenatal COVID-19 Exposure and Neurodevelopmental and Neurological Disorders in Children
Source: JAMA Netw Open. 2026 Sep 3;9(9):e2631847. doi: 10.1001/jamanetworkopen.2026.31847 (PMC13543023; doi:10.1001/jamanetworkopen.2026.31847)
Supplement: Supplementary file 2 [file jamanetwopen-e2631847-s002.pdf]

Data Sharing Statement

Magnus. Prenatal COVID-19 Exposure and Neurodevelopmental and Neurological Disorders in Children. *JAMA Netw Open*. Published September 03, 2026.
doi:10.1001/jamanetworkopen.2026.31847

Data
Data available: No
